# How Technology in Care at Home Affects Patient Self-Care and Self-Management: A Scoping Review

**DOI:** 10.3390/ijerph10115541

**Published:** 2013-10-29

**Authors:** José M. Peeters, Therese A. Wiegers, Roland D. Friele

**Affiliations:** 1 NIVEL, Netherlands Institute for Health Services Research, Otterstraat 118-124, Utrecht 3513 CR, The Netherlands; E-Mails: t.wiegers@nivel.nl (T.A.W.); r.friele@nivel.nl (R.D.F.); 2 Faculty of Social and Behavioural Sciences, Tilburg University, Warandelaan 2, Tilburg 5037 AB, The Netherlands

**Keywords:** self-care, self-management, technology, care at home, scoping review

## Abstract

The use of technology in care at home has potential benefits such as improved quality of care. This includes greater focus on the patients’ role in managing their health and increased patient involvement in the care process. The objective of this scoping review is to analyse the existing evidence for effects of technology in home-based care on patients’ self-care and self-management. Using suitable search terms we searched the databases of Pubmed, Embase, Cochrane Library, Cinahl, Picarta and NIVEL dating from 2002 to 2012. Thirty-three studies (six review studies and twenty-seven individual studies) were selected. Effects were extracted from each study and were classified. In almost all the studies, the concepts self-care and self-management are not clearly defined or operationalized. Therefore, based on a meta-analysis, we made a new classification of outcome measures, with hierarchical levels: (1) competence (2) illness-management (3) independence (social participation, autonomy). In general, patient outcomes appear to be positive or promising, but most studies were pilot studies. We did not find strong evidence that technology in care at home has (a positive) effect on patient self-care and self-management according to the above classification. Future research is needed to clarify how technology can be used to maximize its benefits.

## 1. Introduction

***What is already Known about the Topic?***
  - The use of technology in the care at home has potential benefits, such as improved patient outcomes, increased quality of care and increased patient involvement in the care process.
  - Implementation of technology in the care at home is not always successful and it takes a long time before innovation of promising technology is implemented on a wide scale.
***What Does This Paper Add?***
  - In our review, we found 33 studies (six review studies and twenty seven individual studies) that reported effects of technology in the care at home on patient self-care and self-management.
  - In almost all of the 33 included studies the authors did not use a clear definition or operationalization of self-care and self-management.
  - Most of the included studies show effects of technology on the level of increased patient competence: patients using technology have a better understanding of their disease and more knowledge of the disease.
  - We did not find strong convincing evidence that technology in the care at home has (a positive) effect on patient self-care and self-management.
  - These findings can help to guide future research and clinical practice that support self-management efforts.

Substantial increases in the numbers of chronically ill patients, in the face of an expected reduction of staffing in health care and significant financial problems, mean that a fundamental change is required in the process of care [1]. Faced with rising demand for health care on the one hand and health system capacity constraints on the other hand, governments and care organizations are increasingly turning to new technology to help support and enhance existing services [2].

The main (expected) benefits of technology in care at home are reduced costs from less hospital utilization as well as improved health outcomes among patients with chronic illnesses, most often heart failure, diabetes or other chronic diseases [3]. Technology is being considered by homecare organizations with a view to managing costs and enabling independence for patients wishing to stay at home [4]. These potential benefits are recognised in health policy [2]. The use of technology to provide health services for independently living individuals with chronic diseases and for frail elderly is one of the most promising developments in health care nowadays.

The development and use of technology in care at home is important in the context of the growing emphasis on self-care and self-management in health care. Technology can be used as a tool to monitor symptoms of disease and therefore affords patients the opportunity to manage chronic illness. The need to manage chronic conditions and to actively engage in a lifestyle that fosters health is increasingly recognized as the responsibility of the patient. Optimal self-management entails the ability to monitor one’s illness and to develop and use cognitive, behavioural, and emotional strategies to maintain a satisfactory quality of life [5]. Current evidence also indicates that patients who engage in self-management behaviours improve their health outcomes [6]. Self-management is especially important for those with a chronic disease, because management of the illness is a lifetime task and self-management can enable patients to make their own decisions. For these patients it is also important to learn how to cope with the disease and manage it in their daily life, in their social contacts and in their job. Technology in care at home can be used by patients, for example to monitor their symptoms, and may contribute to patients’ independence, enabling them to stay at home for as long as possible.

The use of technology in care at home is a relatively new research field and the body of evidence regarding its effects is sparse [3]. There is a large gap between the postulated and empirically demonstrated benefits of technology [7]. Moreover, little is known about the effects of promising technology in terms of patient outcomes such as self-care and self-management of an illness. The aim of this review is to analyse the existing evidence for the effects of technology in home-based care on self-care and self-management.

***Technology in Care at Home***
  Technology in care at home is a broad term and there are many different types of technology. We give some examples. Home telemonitoring is defined as an automated process for the transmission of data about a patient’s health status from the patient’s home to the respective health care setting [1,8]. Its aim is to provide information to the health professionals without their having to visit the patient.
  *Home telecare* is focused on providing support from a distance to patients in their own home. *Home telemedicine* is defined as the direct provision of clinical care, including diagnosing, treating or consultation, via telecommunication. This may include the sharing of scans and visual images [1]. The primary function of home telemedicine is to provide specialist consultation to distant communities, rather than offer a tool for self-management of chronic disease. Assistive technology applications are very diverse, and can range from specific alarm and monitoring devices to ambient living technology. These can be used in several ways, for example to increase the comfort and independence of (chronically) ill patients [9]. For this paper, all these described domains of technology in care at home are included.

### 1.1. Conceptualization of Self-Care and Self-Management

A number of authors have put forward definitions of self-care and self-management. Lorig and Holman [10] defined s*elf-management* as a dynamic, interactive and daily process, aimed at helping patients maintain a wellness perspective by engaging in a set of tasks: medical management (maintaining, changing, and creating new meaningful behaviours or life roles) and emotional management (dealing with the emotional consequences of having a chronic condition). *Self-care* is defined as a two-phase process of (1) maintaining health through positive health practice and (2) managing a chronic disease through a process of recognizing, evaluating, and treating symptoms, and evaluating the efficacy of the treatments chosen [11].

Well-known conceptual frameworks have enhanced the understanding of self-care [12] and the understanding of self-management of chronic illness [10]. Riegel and Dickson [12] describe self-care as a naturalistic decision-making process involving the choice of behaviours to maintain physiological stability (maintenance) and the response to symptoms when they occur (management). The self-care process according to Riegel and Dickson [12] has five successive, hierarchical stages: (1) symptom monitoring; (2) symptom recognition; (3) symptom evaluation; (4) treatment implementation; (5) treatment evaluation. Lorig and Holman [10] attempt to give meaning and substance to the term self-management and present six core self-management skills: (1) problem solving; (2) decision making; (3) resource utilization; (4) forming of a patient/health care provider partnership; (5) taking action; (6) self-tailoring.

### 1.2. Research Focus

Although expectations about the impact of health technology on patient self-care and self-management are high, until now the evidence is not clear. This study expands on the growing body of literature on this subject and focuses on the effects of technology in care at home on patient self-care and self-management, irrespective of the specific type of technology. We have not made selections or applied any quality constraints in relation to the technology.

The research question we aim to answer is: “What are the effects of the use of technology in care at home on patient self-care and self-management?” 

The goals of our review are: (1) to analyse the evidence for the effects of technology in the care at home on patient self-care and self-management, without limiting the type of technology used or the target group it is intended for; (2) to identify knowledge gaps in the existing literature.

## 2. Methods

### 2.1. Scoping Review

We conducted a scoping review on the impact of technology in care at home. Scoping reviews represent an increasingly popular approach to reviewing health research evidence [13]. Definitions commonly refer to “*mapping*” a range of evidence in order to convey the breadth and depth of a field [14]. In this study, we used a scoping review because a narrow research question could not be defined [15]. Scoping studies differ from systematic reviews because authors typically do not assess the methodological quality of the included studies [15,16]. Scoping studies also differ from narrative or literature reviews in that the scoping process requires analytical reinterpretation of the literature [13].

### 2.2. Literature Search

A comprehensive literature search was conducted in July 2012. The keywords (*i.e.*, technology, home telecare, telemonitoring, telemedicine, telecommunication, community dwelling, independent living, self-care, self-management, disease-management) were determined after an initial broad search of the literature and consultations by two of the authors (JP, TW) with a librarian. We decided to use a broad search string to make sure we would identify as many relevant studies as possible. The following main databases for the subject of our study were searched:

(1). pubmed (United States National Library of Medicine)
(2). EMBASE (Excerpta Medica Database)
(3). COCHRANE LIBRARY (*Cochrane Database of Systematic Reviews*, Cochrane Central Register of Controlled Trials, Cochrane Methodology Register, Database of Abstracts of Reviews of Effects, Health Technology Assessment Database, NHS Economic Evaluation Database)
(4). CINAHL (Cumulative Index to Nursing and Allied Health Literature)
(5). PICARTA (Dutch Central Catalogue NCC and the Online Contents)
(6). NIVEL (Netherlands Institute for Health Services Research)

Table 1 shows which search terms were used for these databases.

**Table 1:** Search terms.

“*Technology*” *search terms*: Technology, homecare, hometelecare, telecommunications, telemonitoring, telemedicine, teleconsultation, e-health, telehealth, telenursing, smart phone, mobile device, apps, ipad, social media, sms, robotics, remote care, remote sensing, video-communication, e-coaching, mobile health, m-health, gaming, health 2.0, wireless communication, data storage device, computer storage device, mobile device, electronic care, gerotechnology, sensor, camera, webcam, domotica
“*Self-(disease)management/self-care*” *terms*: Self-care, self-management, disease management, independence
“*Care at home*” *terms*: Care at home, home care, assisted living, assisted living facilities, independent living, home bound patients, home bound persons, community dwelling, ambient assisted living, home environment, patients home

### 2.3. Search Terms and Strategy

A combination of free-text and thesaurus terms were used. “Technology” search terms were combined with “self-care/independence” or “self-(disease) management” search terms and with “care at home” search strings (see Table 1 for details). In addition to the searches in the literature databases, reference lists of included systematic literature studies (e.g., [1,17]) were screened. All references tracked down were entered in Reference Manager, where double entries were removed. We also removed publications without abstract and publications that did not provide research results, such as opinion letters, book chapters, commentaries, models or proposals for research.

### 2.4. Inclusion and Exclusion Criteria

We applied limitations for:

(1) year of publication: only from 2002 to 2012;
(2) language: published in the English language;
(3) type of publication: appearing in peer-reviewed journals;
(4) country: only Western, Anglo-Saxon or Asian countries.

### 2.5. Inclusion Criteria

The following inclusion criteria were used:

- The study is aimed at technology for patients living at home (sheltered housing, e.g., for persons with disabilities, was also included).
- The publication documents outcome effects of technology, *i.e.*, patients self-management, disease management, self-care or independence.

No criteria for patient groups or research designs were applied. Publications about the same study were considered as one study, for analytical purposes.

### 2.6. Exclusion Criteria

The following exclusion criteria were used:

- Studies that focussed exclusively on the technology itself, or on technology that is used only by care providers.
- Studies in which technology features, but is not the focus of the study, for example studies about technology-dependent children.

The references found were assessed in two steps, to ascertain whether they were eligible for inclusion. The first step involved the title and the abstract; the second step concerned the full text. The assessment was based on content-analysis and performed by two reviewers (JP, TW), independently of each other. Disagreements between the reviewers on whether to include or exclude a publication were discussed until consensus was reached.

### 2.7. Data Extraction and Synthesis

A spread sheet was created to chart the information that contributed to answering the research question. Subsequently, the following data from the included studies were extracted: year of publication, country where the research was conducted, number of patients involved in the study, patient category (e.g., chronic disease), technology used and observed effects. The data extraction was conducted by one reviewer (TW). The information extracted for the purpose of answering the research questions was checked by a second reviewer (JP). The results were evaluated in a narrative format to provide a detailed summary and comparison of the technology used in care at home, across the reported outcome measures of our review.

### 2.8. Search Results

**Figure 1:**
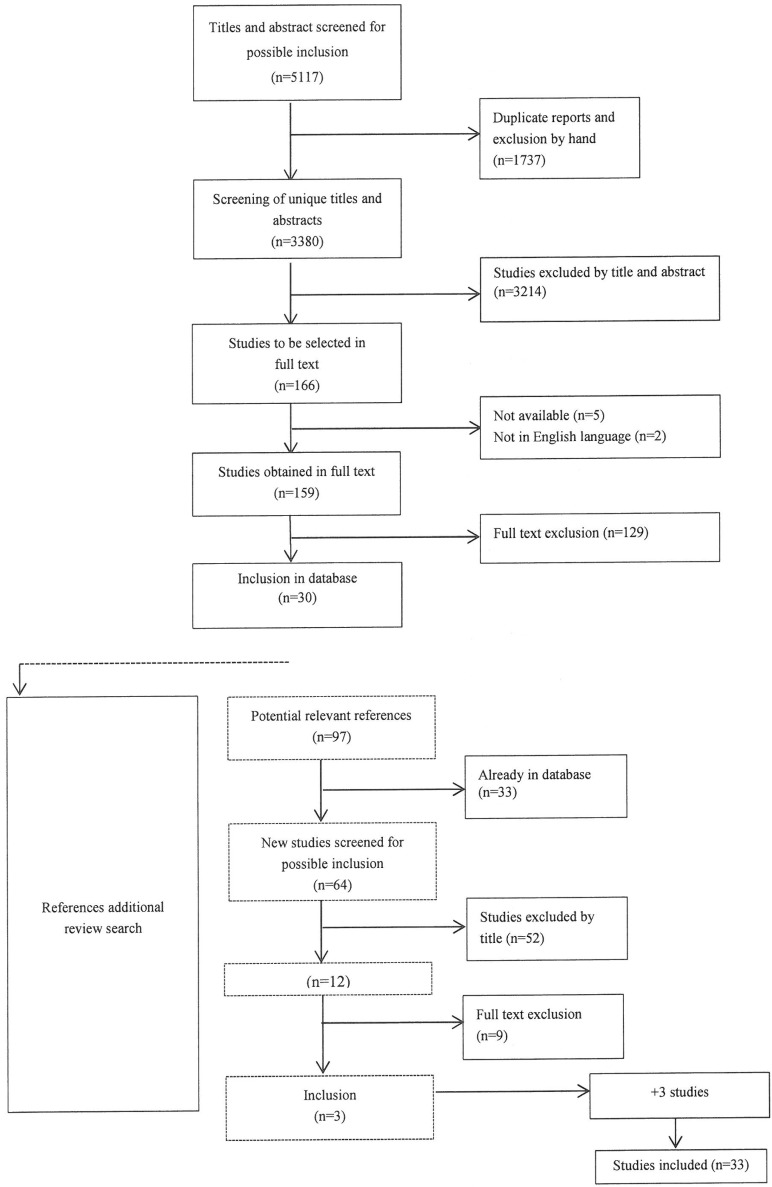
Flow chart of study inclusions.

The initial search actions resulted in 5,117 hits in total. After removing duplicate articles and articles without an abstract, 3,380 references remained. The inclusion flow is depicted in Figure 1.

After selecting references by title and abstract, there were 159 left to be judged for inclusion based on their full text. Most of these studies were excluded, based on the inclusion and exclusion criteria (see above). In this phase, 30 studies remained. Screening of the references contained in the review studies yielded another 64 possibly relevant references that were not already included in our database. Ultimately, 33 studies (6 review studies and 27 individual studies) were selected for inclusion for data extraction and analysis and provided the main empirical evidence base in relation to assessing the effects of technology on patient self-care and self-management in care at home.

**Table 2:** Study characteristics and results.

Author and year of publication	Country	Type of patients	Type of study	Sample	Type of technology
**Reviews**					
Paré *et al*., 2007 [1]	Canada	chronic diseases	systematic review	65 studies, number of patients not mentioned	telemonitoring: the use of audio, video and other telecommunication technologies to monitor patient status at a distance
Bowles & Baugh, 2007 [2]	USA	adult patients with chronic illness	summary of publications	19 studies (28 papers)	telehomecare: telehealth technology with peripheral medical devices
Stumbo *et al*., 2009 [17]	USA	individuals with disabilities	literature and research synthesis	71 studies, number of patients not mentioned	assistive technology
Gately *et al*., 2008 [18]	UK	patients with long-term conditions	synthesis of qualitative studies	12 studies, 253 patients	health technologies at home
Jaana & Paré 2007 [19]	Canada	diabetes	literature review	17 studies, 1,535 patients	telemonitoring: transmission and remote interpretation of patients’ data
Jaana *et al*., 2007 [20]	Canada	hypertension	literature review	14 studies, 1,119 patients	telemonitoring: automated timely transmission of data, without involvement of health professionals
**Individual studies**					
Gomez *et al*., 2002 [21]	Spain	diabetes	collecting data via patient unit	10 patients	telemedicine system: blood glucose readings downloaded in patient unit
Bujnowska-Fedak *et al*., 2011 [22]	Poland	diabetes	monitoring at home, patient questionnaires	100 patients (50 intervention group, 50 control group)	telehome diabetes monitoring and treatment
Bowles & Dansky, 2002 [23]	USA	diabetes	patient questionnaires and care providers scores	174 patients (84 intervention group, 90 control group)	telehomecare: video visits
Frühauf *et al*., 2012 [24]	Australia	psoriasis patients	patient and provider questionnaires	10 patients	teledermatology: mobile phone with built-in camera for wireless transmission
Finkelstein *et al*., 2008 [25]	USA	multiple sclerosis	patient questionnaires	12 patients	home automated telemedicine
Pecina *et al*., 2011 [26]	USA	complex medical illnesses	qualitative telephone survey	20 patients	telemonitoring: remote monitoring of health parameters, videoconferencing
Marziali, 2009 [27]	Canada	chronic disease	patient interviews	18 patients	protected website with links to e-mail-addresses, discussion forum, videoconferencing
Kuo *et al*., 2012 [28]	Taiwan	stroke patients	in-home monitoring	84 patients	telehealthcare: 24-h tracing and monitoring system of health status and care use
LaFramboise *et al*., 2009 [29]	USA	heart failure	patient focus groups and interviews	13 patients	Health Buddy: device attached to a telephone line, asking 7 questions daily, followed by educational “pearl”.
Dansky *et al*., 2008 [30]	USA	heart failure	patient telephone interview, Self-Care of Heart Failure Index	284 patients	videobased, interactive telehealth system
Bowles *et al*., 2010 [11]	USA	heart failure	patient interviews	188 patients	telehomecare equipment including videophone and wireless devices
Finkelstein & Wood, 2011 [31]	USA	heart failure	patient self-test, survey and interview	10 patients	home automated telemedicine
Papasifakis & Vanderveen, 2009 [32]	USA	heart failure	patient surveys	85 patients	self-monitoring through the use of telehealth
Guendelman *et al*., 2002 [33]	USA	children with paediatric asthma	Health buddy, diary	134 patients (66 intervention group, 68 control group)	Health buddy
Brennan *et al*., 2010 [34]	USA	patients with chronic cardiac disease	patient questionnaires	282 patients (146 intervention group, 136 control group)	technology-enhanced practice
Vontetsianos *et al*., 2005 [35]	Greece	COPD patients	monitoring at home, patient questionnaires	18 patients	telehealth services: transmission of health data, videoconference
Sicotte *et al*., 2011 [36]	Canada	COPD patients	patient and care providers questionnaires	46 patients (23 intervention group, 23 control group)	telemonitoring: web phone with touch-screen monitor, to enter and send data, receive feedback on predetermined parameters and send warnings to nursing staff
Wilson *et al*., 2009 [37]	USA	people aging with disability	home interviews and telephone contacts	91 patients (47 intervention group, 44 control group)	assistive technology, home modifications, adjusted task performance
Shea & Chamoff, 2012 [38]	USA	patients with chronic conditions	secondary analysis of patient and care provider survey data	43 patients	telemonitoring: data collection, knowledge transfer and (asynchronous) communication
Cardozo & Steinberg, 2010 [39]	USA	patients with chronic conditions following discharge	monitoring at home, patient questionnaires	851 patients	case-managed telemedicine: remote monitoring of health status, electronic patient record and Health Buddy
Hoenig *et al*., 2003 [40]	USA	disabled elderly	patient survey, interviews	2,368 patients	technological assistance
Chumbler *et al*., 2004 [41]	USA	frail elderly men	monitoring at home, patient questionnaires	226 patients (111 intervention group, 115 control group)	distance monitoring technology: Health Buddy, two-way audio-video with or without biometric monitoring
Hui *et al*., 2006 [42]	China	older women with urinary incontinence	questionnaires and focus group	58 patients (27 intervention group, 31 control group)	telemedicine: videoconferencing
Bewernitz *et al*., 2009 [43]	USA	dementia	observing three self-care tasks	11 patients	intercom, remote camera, pre-recorded voice, synthesized voice, visual prompts
Evans *et al*., 2011 [44]	UK	dementia	patient questionnaires, semi-structured interviews	1 patient	enabling smart technology: sensors and verbal messages
Mihailidis *et al*., 2008 [45]	Canada	dementia	score sheet, video	6 patients	COACH system: tracking and prompting system
Maguire *et al*., 2005 [46]	UK	cancer patients receiving chemotherapy	grading system, patient questionnaires and interviews	10 patients (4 intervention group, 6 control group)	handheld computer to monitor symptoms

### 2.9. Study Characteristics

Table 2 shows, for each study, the authors(s) and year of publication, the country where the research was conducted, the patient category (e.g., patients with chronic diseases such as diabetes or heart failure), type of study (literature review or individual study), the number of patients involved (in the case of randomized controlled trials: number of patients in the intervention group and control group) and a brief description of the type of technology concerned.

Most studies eighteen of the 33 were conducted in the USA; six in Canada; one in Australia; and three in the United Kingdom (UK). The other studies were conducted in Asian (Taiwan, China) or European countries (Greece, Poland, Spain). The oldest publications date from 2002 [21,23,33] and the most recent from 2012, the year in which the literature search was conducted [24,28,38]. Six studies can be characterised as a (systematic) literature review or synthesis of qualitative studies, and the number of studies covered in these reviews ranged from 12 to 65. The other 27 studies were individual in nature and the size of the research samples in these individual studies ranged from 1 to 2,368 patients (an average of 190 patients). Nine studies can be characterized as a randomized controlled trial (RCT) with one or more follow ups (see Table 2 for more details).

Patient characteristics differed considerably from one study to another: sometimes the intervention group was a select group, such as patients with diabetes, hypertension, heart failure, lung disease or dementia. Other studies were about chronic patients in general, people with disabilities, or frail or disabled elderly. Due to the broad search criteria, the types of technology used are also very different between the studies: telemonitoring, telehomecare, telemedicine, teledermatology, videoconferencing or assistive technology. Studies varied in duration from eight weeks to two years. Clearly, there is a wide range in terms of patient populations, type of technology and type of intervention.

## 3. Results and Discussion

### 3.1. Effects of Technology on Self-Care and Self-Management

Generally, the reported effects of technology use in care at home on self-care and self-management are positive or promising for the near future. This can be concluded both from the review studies and the individual studies. For example, a review of research evidence for the effects of telehomecare on patients with chronic diseases shows that technology appears to have positive effects on chronic illness outcomes, such as self-management, rehospitalizations and length of hospital stay [3]. In a systematic review of observed effects, close management of diabetic patients through telemonitoring showed a significant reduction in complications, good receptiveness by patients and improved patient empowerment and education [19]. A review of hypertension home telemonitoring presents preliminary evidence of the benefits of telemonitoring as a successful self-management approach [20]. Another systematic review of home telemonitoring of chronic diseases found it to be a promising self-management approach that produces accurate and reliable data, empowers patients, influences their attitudes and behaviours, and potentially improves their medical conditions [1].

### 3.2. Lack of Conceptual Clarity

The effects of technology on self-care and self-management appear to be promising for the future. But in a further analysis of the studies in our review, we found serious limitations to the interpretation of the reported results regarding effects of technology on self-care and self-management. The first striking element of the included studies on patient self-care and self-management is the use of varied and inconsistent terminology: self-care, self-management, self-monitoring, self-regulation, adherence and compliance reveal a confused picture. The terms self-care and self-management are often used interchangeably or simultaneously, sometimes referring to knowledge or awareness, in other cases meaning maintaining health and managing a chronic illness. The need for conceptual clarity is not new. Already in 2003, authors started a discussion towards clearly defining self-management and its role in the delivery of health care to people with chronic disease. This lack of conceptual clarity is also discussed and described by Wilson *et al*. [47] in a study on self-management in long-term conditions. In comparing and contrasting definitions of self-care and self-management Wilson *et al*. [47] noted that definitions of self-management are more specific than those of self-care, although there are several common features. Both self-care and self-management involve a proactive process, compliance with professional advice, close attention to one’s body, and having the appropriate coping behaviour. Wilson *et al*. [47] argue that the key difference between self-management and self-care is that in self-management patients undertake tasks that are within the traditional preserve of health professionals. Moreover, Song [48] reported that, despite the increased interest in self-care, there is no clear consensus among researchers and practitioners as to exactly how the concept of self-care should be defined. Finally, Schulman-Green *et al*. [49] described self-management as a dynamic process in which individuals actively manage a chronic illness. In a meta-synthesis of current descriptions of the self-management process, Schulman-Green *et al*. [49] identified three categories of self-management processes: (a) focusing on illness needs; (b) activating resources and (c) living with a chronic illness. The authors delineated tasks and skills for each category of self-management.

Accordingly, a significant deficiency in almost all the studies under review is that the authors do not refer to the current definitions and conceptualisation of self-care and self-management. Furthermore, the reported effects in most studies only relate to one or more aspects of self-care and self-management.

### 3.3. Meta-Synthesis of the Concepts Self-Care and Self-Management

Because we wanted to gain more insight into the concepts of self-care and self-management as described in the studies under review, we performed a meta-synthesis of the 33 studies. The goal of this exercise was to obtain a better understanding of what is meant by these concepts. When we examined the studies of our literature review in greater detail (see Table 3), we discovered that the described effects on self-care and self-management covered a number of different aspects. 

**Table 3:** New classification of patient outcome measurements.

Author and year of publication	Type of patient	Competence	Illness-management	Independence
**Reviews**				
Paré *et al*., 2007 [1]	chronic diseases	improvement of awareness and feeling of security, leading to empowerment active participation in the process of care		
Bowles & Baugh 2007 [2]	adult patients with chronic illness		positive effects on self-management	
Stumbo *et al*., 2009 [17]	individuals with disabilities	more control more self confidence		assistive technology is a foundational support that produces multiple and life-changing benefits
Gately *et al*., 2008 [18]	patients with long-term conditions	disruptive effects of health technologies on personal identities more self-regulation	disruptive effects of health technologies on strategies of managing illness	
Jaana & Paré, 2007 [19]	diabetes	receptiveness, empowerment, education	management of medical condition	
Jaana *et al*., 2007 [20]	hypertension	significant reduction in blood pressure, significant improvement of disease knowledge		
**Individual studies**				
Gomez *et al*., 2002 [21]	diabetes	increasing patient empowerment and education		
Bujnowska-Fedak *et al*., 2011 [22]	diabetes	achieving a sense of independence		
Bowles & Dansky, 2002 [23]	diabetes	improved knowledge (not significant)	improved self-management	
Frühauf *et al*., 2012 [24]	psoriasis patients	more flexible and empowered lifestyle		
Finkelstein *et al*., 2008 [25]	multiple sclerosis		improvement of functional outcomes	
Pecina *et al*., 2011 [26]	complex medical illnesses	moderate increase in knowledge earlier detection of decline in health status increased personal awareness leading to behavioural changes		
Marziali, 2009 [27]	chronic disease	reduced sense of isolation, maintenance of optimal healthcare strategies		
Kuo *et al*., 2012 [28]	stroke patients		reduce daily abnormal blood pressure rate by proper measurement	
LaFramboise *et al*., 2009 [29]	heart failure		ease of use, promote comprehension and self-management	
Dansky *et al*., 2008 [30]	heart failure	confidence is a predictor of self-management behaviours		
Bowles *et al*., 2010 [11]	heart failure		early identification of and intervention in clinical changes	
Finkelstein & Wood, 2011 [31]	heart failure		assumed utility in daily self-management	
Papasifakis & Vanderveen, 2009 [32]	heart failure		improvement of self-care improvement of disease management	
Guendelman *et al*., 2002 [33]	paediatric asthma	self-care behaviours improved far more for the intervention group; increased self-management skills		improved asthma outcomes
Brennan *et al*., 2010 [34]	patients with chronic cardiac disease		better self-management	improved outcome
Vontetsianos *et al*., 2005 [35]	COPD patients	improvement of disease knowledge	improvement of self-management	
Sicotte *et al*., 2011 [36]	COPD patients	improving attitudes and behaviours concerning management of the illness		
Wilson *et al*., 2009 [37]	people aging with disability		reducing or slowing down functional and frailty problems	improved ability to gain or maintain independence
Shea & Chamoff, 2012 [38]	patients with chronic conditions	improved self-care behaviour		
Cardozo & Steinberg, 2010 [39]	patients with chronic conditions following discharge	improved disease understanding		
Hoenig *et al*., 2003 [40]	disabled elderly		technological assistance may be substituted for some personal assistance	
Chumbler *et al*., 2004 [41]	frail elderly men			improvement in functional and cognitive outcomes
Hui *et al*., 2006 [42]	older women with urinary incontinence		videoconferencing is as effective as conventional management	
Bewernitz *et al*., 2009 [43]	dementia	increased independence in some tasks		
Evans *et al*., 2011 [44]	dementia			potential tool to support independent living
Mihailidis *et al*., 2008 [45]	dementia			improvement in independence
Maguire *et al*., 2005 [46]	cancer patients receiving chemotherapy		improving symptom management	

We also found that most of the reported effects were related to the improvement of disease knowledge or education, increased self-management skills, such as symptom management, and the improvement of attitudes and behaviours concerning management of the illness. We also discovered that these concepts contained a number of components, identified as, for instance: tasks, skills, self-management behaviour and self-management processes. This analysis continued until a final categorization was reached.

### 3.4. New Classification of Outcome Measures

Based on this knowledge, we made a new classification of outcome measures, with three hierarchical levels or stages:

(1) competence (a better understanding of the disease, disease knowledge);
(2) illness management (making choices, acting responsibly);
(3) independence (social participation, autonomy).

At the first stage, there is an increase in patient awareness of the disease and an increased knowledge of the symptoms; at this level, patients need the help of professionals to manage their disease. At the second stage, patients are increasingly involved in the care process, are able to manage the disease, can make important decisions, e.g., about the treatment, and the professional becomes a co-pilot. At the highest stage, patients are fully able to take care of themselves, participate in work or their social network, and live independently. The professional is out of sight.

We applied this tripartite division to the described effects and outcome measures of our literature review. For each study we looked in detail at the meaning of the described effects and attached a new label to the measurements, namely *competence*, *illness management* and *independence*. The results of this exercise are presented in Table 3. This new classification can be used to determine the stage of a patient’s self-care or self-management process and what sort of skills and tasks are needed.

### 3.5. Discussion

This review aims to afford insight into the effects of technology in care at home on patient self-care and self-management by critically evaluating the literature on this topic. Although this is a relatively new research field, we found 33 studies with a focus on effects on patient outcomes. The huge diversity in research methods in the included studies reflects the newness of this research field.

Although almost all the authors in our review reported positive effects of technology on self-care and self-management, and underline the importance of promising technology, we did not, however, find strong evidence for these positive effects. Part of this is caused by the lack of a clear conceptualization of self-care and self-management. This is in line with the findings of Song [48] and Schulman-Green [49].

Most of the studies showed effects of technology in home-based care on *competence* level: in general, patients using certain types of technology have a better understanding of their disease and more knowledge about the disease. Also there are reported effects on *illness management*: patient self-care behaviour improved and patients were more capable of managing their medical condition and their illness. Only a few studies showed an effect on the highest level of our classification, namely *independence*: patients showing an improved ability to gain or maintain more independence or to live more independently using technology. We therefore conclude that the evidence for positive effects of technology in care at home on self-care and self-management, according to our tripartite classification, is not strong.

### 3.6. Methodological Quality of the Studies

A critical comment on the results of the studies under review concerns methodological quality. Besides the lack of clarity about the concepts self-care and self-management, an important shortcoming of many of the studies is that they consisted of pilot projects with relatively small numbers of patients, and were limited to specific patient groups (see Table 2). Furthermore, the duration of the interventions was relatively short—usually one year. Some studies had only one follow-up assessment. As a result, the magnitude of the described effects of technology on patients self-care and self-management is debatable, because of the variation in patients’ characteristics (e.g., background, ability for self-management), small sample sizes with various types and doses of intervention, inconsistent selection of samples and different approaches to intervention and control groups. 

Further, the research designs of the studies were very diverse. They included qualitative research, monitoring studies, cross-sectional research, single case studies and randomized controlled trials. In addition, only a third of the individual studies (nine studies) used both an intervention group (patients who use technology) and a control group (patients who received usual care). Concerning the methodology, most of the studies used patient questionnaires or patient (telephone) interviews.

### 3.7. Strengths and Limitations

The broad scope of our review is its main strength. Most of the studies on the effects of technology on patient self-care and self-management focus on one disease, a specific patient group or a particular setting. We brought the results together and gave an overview of the results of the thirty-three studies, including six review studies. This broad scope also has its drawbacks, namely in capturing the breadth of self-care and self-management more than the depth. Nonetheless, in the meta-analysis, we tried to reach a better understanding of self-care and self-management and to give more insight into the meaning and complexity of the concepts.

### 3.8. Knowledge Gaps

Self-care and self-management are often-used concepts, but to date there has been no agreement about their definition and operationalization in the literature. More effort is required to further operationalize the meaning of these concepts. Self-care and self-management processes include tasks, skills, and competences aimed at coping with the illness and integrating the illness in the context of an individual life. Research on the effects of technology in care at home is still in its infancy. Researchers should learn from the current body of knowledge in this area and address the issues of methodological quality of the studies that remain problematic. There is a lack of high quality evaluation research, and further evaluation studies should consider strong research designs, with a control group, with larger samples of patients and conducted over longer periods of time. Furthermore, we found that the use of validated instruments to measure effect on self-care and self-management was limited. But it is not only large scale research designs that are needed. Indeed, randomized control studies are not always the most appropriate in this relatively new research field. Qualitative research and realistic field studies are also important to gain more insight into the self-care and self-management process.

Future research is needed to clarify how technology can be used in care at home to maximize its benefits, to examine how and when patients engage in self-care and self-management, under what conditions, and to identify differences in self-care and self-management in patient characteristics, in specific diseases and in various settings. Only then will we be able to draw firmer conclusions regarding the effects of technology.

## 4. Conclusions

In this scoping review we did not find convincing evidence that technology in care at home has (a positive) effect on patient self-care and self-management. The reported effects are mostly concerned with competence, self-care behaviour and illness management. Less is known about the contribution of technology in care at home to helping patients remain independent. In general, patient outcomes in the included studies were positive, but most of the studies were pilot studies, with a small number of patients, had no control group and a short duration with only one follow-up assessment.

Management of a chronic disease and identification of the components of self-care and self-management are important for health care. These findings can help to guide future research and clinical practice that support self-management efforts.
